# DNA damage and apoptosis induced by a potent orally podophyllotoxin derivative in breast cancer

**DOI:** 10.1186/s12964-018-0263-9

**Published:** 2018-09-03

**Authors:** Yajie Wang, Hua Sun, Zhiyan Xiao, Gang Zhang, Dan Zhang, Xiuqi Bao, Fangfang Li, Shaoyu Wu, Yuanchao Gao, Ning Wei

**Affiliations:** 1 State Key Laboratory of Bioactive Substance and Function of Natural Medicines, Institute of Materia Medica, Chinese Academy of Medical Sciences and Peking Union Medical College, Beijing, 100050 People’s Republic of China; 2 Institute of Chinese Materia Medica, China Academy of Chinese Medical Sciences, Beijing, China; 3 Guangdong Province Key Laboratory of New Drug Screening, School of Pharmaceutical Science, Southern Medical University, Guangzhou, China; 4 Beijing Tsinghua Changgeng Hospital, Beijing, China; 5 Division of Hematology-Oncology, Department of Medicine, University of Pittsburgh School of Medicine, Pittsburgh, PA USA; 6 Cancer Therapeutics Program, University of Pittsburgh Cancer Institute, University of Pittsburgh, Pittsburgh, PA USA

**Keywords:** Breast cancer, TopoisomeraseII, DNA damage, p53, Mdm2

## Abstract

**Background:**

Targeting TopoisomeraseII (TopoII) and generate enzyme mediated DNA damage is an effective strategy for treatment of breast cancer. TopoII is known as a validated target for drug discovery and cancer chemotherapy.

**Methods:**

XWL-1-48, a new orally podophyllotoxin derivative, was designed and synthesized. The effect of XWL-1-48 on TopoII binding and activity was determined by molecular docking software and kDNA-decatenation assay, respectively. In vitro and in vivo breast cancer models were used to document the antitumor activity of XWL-1-48. Cellular apoptosis, cell cycle and ROS were analyzed by flow cytometry. Alteration of XWL-1-48-mediated downstream pathways was determined by western blot analysis.

**Results:**

The cytotoxicity of XWL-1-48 is more potent than that of its congener GL331. Molecular docking demonstrated that XWL-1-48 could bind to TopoII through forming two strong hydrogen bonds and potential pi-pi interactions. Noticeably, XWL-1-48 exerts potent antitumor activity in in vitro and in vivo breast cancer model. Treatment with XWL-1-48 caused ROS generation and triggered DNA damage through induction of γ-H2AX and activation of ATM/p53/p21 pathway. Further studies showed that XWL-1-48 led to S-phase arrest and mitochondrial apoptosis. Meanwhile, XWL-1-48 significantly blocked PI3K/Akt/Mdm2 pathway and enhanced Mdm2 degradation.

**Conclusion:**

XWL-1-48 may be a promising orally topoII inhibitor, its mechanisms are associated with suppression of TopoII, induction of DNA damage and apoptosis, blockage of PI3K/AKT/Mdm2 pathway.

## Background

Currently, breast cancer is the most common cancer type diagnosed and the second leading cause of cancer death in women [1]. It is predicted up to 3.2 million new cases will be diagnosed worldwide each year by 2050 [2]. Although much progress has been made in breast cancer treatment, there are remaining some barriers to cure for breast cancer, including intratumor genomic heterogeneity, lack of efficient predictive biomarkers, acquired multidrug resistance, and so on [3–6]. Therefore, it is urgent to design and develop novel and effective therapies for the treatment of breast cancer.

Noticeably, targeted DNA damage and DNA repair is a successful strategy for treatment of breast cancer. TopoisomeraseII (TopoII) is known as a validated target for drug discovery and cancer chemotherapy. The DNA TopoII plays a key role in the process of transcription, replication, and chromosome segregation [7]. There are two isoforms of TopoII, α and β forms. TopoIIα is essential for the proliferation of growing cells and able to decatenate the replicated chromosomes during the process of chromosome segregation [8]. Thus, the expression level of TopoIIα is significantly upregulated during cell proliferation. Furthermore, the expression level of TopoIIα changes over the cell cycle and reaches a maximum point in the late S and G2/M phase. Moreover, TOP2A (gene of DNA topoisomerase 2-alpha) expression in breast cancer was associated with high proliferation and aggressive tumor subtypes and appears to be independent of its amplification status [9].

TopoII inhibitors are classed into two groups: catalytic inhibitors and poisons. Catalytic inhibitors prevent the formation of the cleavage complex through inhibition of TopoII binding caused by its intercalation into DNA [10]. For example, bisdioxopiperazines and ICRF-187 are catalytic inhibitors that stabilize the closed clamp intermediate, which are formed by the enzyme around the DNA, and blocks ATP hydrolysis [11]. In contrast, TopoII poisons stabilize the cleavage complex, and can be categorized as interfacial or covalent [12]. For example, etoposide (VP16), doxorubicin and mitoxantrone as the interfacial poisons non-covalently bind to the cleavage complex. While quinones, isothiocyanates, and maleimides as covalent poisons have protein reactive groups that form adducts with the enzyme. The stabilization of the DNA cleavage complex results in the formations of permanent double strand breaks [13, 14].

**Fig. 1:**
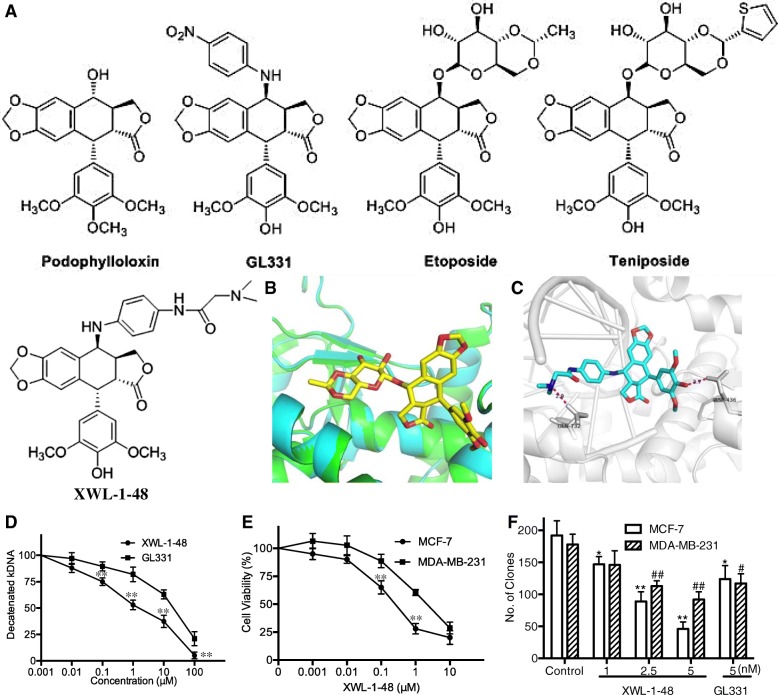
Effect of XWL-1-48 on TopoII activity and growth of breast cancer cells. **a** The chemical structure of podophylloloxin, GL331, Etoposide (VP16), Teniposide and XWL-1-48 is shown; **b** Superimposition of Top2α homology model (cyan) and Top2β crystal structure (Green), shown in cartoon representation. XWL-1-48 pose from the Top2β crystal structure (stick representation, carbons yellow) shown for clarity; **c** Molecular docking study of XWL-1-48 with carbons in cyan to crystal structure of Top2α-DNA homology model. Only interacting residues were labeled. Hydrogen bonds are shown as purple dotted lines; **d** To determine the effect of XWL-1-48 on TopoII activity, kDNA decatenation assay was performed as described in materials and methods; **e** The cytotoxic effect of XWL-1-48 on growth of MCF-7 and MDA-MB-231 cells was determined by MTT assay. IC_50_ was calculated by Prism6.0 software; **f** MCF-7 or MDA-MB-231 cells were seeded in a 6-well plate, and incubated with XWL-1-48 (1, 2.5, 5 nM) or GL331 (5 nM) for 24 h. After 10–14 days, colonies (> 50 cells) were fixed and manually counted. A representative of three experiments is shown. Data were shown as mean ± SD of three independent experiments. *p < 0.05, **p < 0.01 vs. control

VP16, teniposide (VM26) and GL331 are semisynthetic derivatives of podophyllotoxin which targeted inhibition of TopoII activity. They are currently used clinically for treatment of various types of cancer, including breast cancer [15, 16]. They appear to act by causing breaks in DNA via an interaction with DNA TopoII or by the formation of free radicals. However, there is still several limitations such as poor water solubility, metabolic inactivation and development of drug resistance [17]. Therefore, we are trying to design and develop new derivatives of podophyllotoxin that could overcome these deficiencies. Herein, XWL-1-48 (Fig. 1a), a new podophyllotoxin derivative, as oral TopoII inhibitor was designed and synthesized. In vitro and in vivo antitumor activity of XWL-1-48 was evaluated in breast cancer model. Furthermore, the precise mechanism of XWL-1-48 against breast cancer is investigated.

## Methods

### Drugs and chemicals

A new derivative of podophyllotoxin, XWL-1-48, was synthesized by Dr. Xiao’s lab. The purity of XWL-1-48 is more than 98% (HPLC). The chemical structure is shown in Fig. 1a. It is freshly dissolved in dimethyl suifoxide (DMSO) before use. The final concentration of DMSO is less than 0.1% in all the experiments. GL331 also provided by Dr. Xiao. LY294002 (a pan-PI3K inhibitor), 1-(4, 5-dimethylthiazol-2-yl)-3, 5-diphenyformazan (MTT) and other chemicals were purchased from Sigma chemical Co. (St. Louis, MO).

### kDNA-decatenation assay

kDNA-decatenation assay was performed as previously described [18]. In brief, the standard reaction mixture consisted of assay buffer (3 μL of 10× buffer per assay), ATP (1 μL), kDNA (2 μL) and water. 3 μL of XWL-1-48, GL331 or 0.1%DMSO was added into the reaction mixture. After that, 3 μL enzyme was added and incubated for 30 min at 37°C, the reaction was stopped by adding 30 μL of STEB and 30 μL of chloroform/isoamyl alcohol (v:v, 24:1). The result was detected by agarose gel analysis.

### Cell viability assay

MCF-7 and MDA-MB-231 cells were grown in DMEM (GIBCO) medium supplemented with 10% heat-inactivated newborn calf serum, 100 U/mL penicillin, and 100 μg/mL streptomycin. Cell viability was determined by MTT assay. As our previous described [19], cells were seeded in 96-well plates. After an overnight incubation (37 °C with 5% CO_2_), various concentrations of XWL-1-48 was added into wells and incubated for additional 72 h. Thereafter, 100 μL of 0.5 μg/mL MTT was added to each well after withdraw the culture medium and incubated for an additional 4 h. The resulting formazan was dissolved in 150 μL DMSO after aspiration of the culture medium. Plates were placed on a plate shaker for 30 min and read immediately at 570 nm using a micro-plate reader (Bio-Rad Model 450). The IC_50_ was determined in duplicates and each experiment was repeated 3–5 times under identical conditions. IC_50_ value was calculated by Graphpad Prism 6.0 software.

### Clonogenic assay

MCF-7 and MDA-MB-231 cells were seeded in 6-well plates at density of 300 cells/well. On the following day, cells were exposed to various concentrations of XWL-1-48 (1, 2.5, 5 nM) or GL-331 (5 nM) for 24 h. After that, the growth medium was then replaced with fresh medium. After 10–14 days, cell colonies were fixed with trypan blue solution (75% methanol/25% acetic acid/0.25% trypan blue) for 15 min, washed with PBS twice, and air-dried before counting colonies > 50 cells.

### Flow cytometry analysis

#### Cell cycle

MCF-7 or MDA-MB-231 cells were seeded into 6-well plates at density of 4.0 × 10^5^. After exposed to XWL-1-48 (1, 3, 10 μM) or GL-331 (10 μM) for 24 h, the cells were collected, fixed in 70% ice-cold ethanol, and stored at 4 °C overnight. To determine cell cycle distribution, the cells were transferred into PBS, incubated with RNase A (50 mg/ml) for 30 min at 37 °C, followed by 30 min treatment with propidium iodide (PI, 50 mg/ml) at 37 °C. The cells were washed and resuspended in PBS. The fluorescence levels were analyzed by flow cytometry (Beckman Coulter, USA).

#### Apoptosis

Briefly, following 24 h treatment with XWL-1-48 (1, 3, 10 μM) or GL-331 (10 μM), MCF-7 cells were resuspended with the cold binding buffer. According to the manufacture’s instruction (KeyGEN Biotech Inc., China), 5 μL of Annexin-V-FITC and 5 μL of PI were added and the cells were incubated for 10 min in dark at room temperature. Flow cytometry analysis was performed using a FACS (Beckman coulter, USA).

#### Measurement of ROS generation

The levels of ROS (Reactive Oxygen Species) were measured by DCFH-DA which is a freely permeable tracer specific for ROS. DCFH-DA can be deacetylated by intracellular esterase to the non-fluorescent DCFH which is oxidized by ROS to the fluorescent compound 2′, 7′-dichloroflorescein (DCF). Thus, the fluorescent intensity of DCF is proportional to the amount of ROS produced by the cells [20]. 1 × 10^5^ MCF-7 cells/well were exposed to XWL-1-48 or GL331 for 24 h and 1 mM H_2_O_2_ used as a positive control. Then, cells were harvested, rinsed twice with PBS and incubated with DCFH-DA (10 μM) in the dark at 37 °C for 30 min. The cells were washed and resuspended in PBS. The cell-associated fluorescence was measured with FACS (Beckman Coulter, USA).

#### Mitochondrial transmembrane potential (DCm) measurement

The DCm was analyzed by JC-1 Mitochondrial Membrane Potential Assay Kit (KeyGEN Biotech Inc., China). JC-1 (5, 5′, 6, 6′- tetra-chloro-1,1′,3,3′-tetra-ethylbenzimidazol-carbocyanine iodide) is capable of selectively entering mitochondria, where it forms aggregates and emits red fluorescence when DCm is high. At low DCm, JC-1 cannot enter into mitochondria and forms monomers emitting green fluorescence. The ratio between green and red fluorescence provides an estimate of changes in the mitochondria membrane potential (DCm). MCF-7 cells were treated with desired concentrations of XWL-1-48 for 24 h. After trypsinisation and PBS washing, 1 × 10^5^ cells were incubated for 20 min in freshly prepared JC-1 (1 mM) solution at 37 °C. Spare dye was removed by dye buffer solution washing. The cell-associated fluorescence was measured with FACS (Beckman Coulter, USA).

### RT-PCR analysis

MCF-7 cells were seeded in 6-well plates at a density of 2 × 10^5^ cells and allowed to attach overnight, then cultured with XWL-1-48 (0, 1, 3, 10 μM) for 24 h. Total RNA was extracted by the guanidine isothiocyanate/phenol/chloroform method. The integrity and purity of the RNA were checked by UV Spectrophotometer for OD260 and OD280, then reverse transcripted from mRNA to cDNA using the RT-PCR kit (Promega, WI, USA). The following primers were used for RT-PCR: MDM2 (509 bp) sense 5′- AGA AGG TTC TGG GAA GA TCGC-3′, anti-sense 5‘- GTT GAT GGC TGA GAA TAG -3’; β-actin (529 bp) sense 5‘- CTT GAT GCT GGT GTA AGT-3’,anti-sense 5‘-AGC ACT GTG TTG GCG TAC AG-3’. The PCR profile was as follows: 10 min at 95 °C, followed by 30 cycles of 30 s at 95 °C and 1 min at 60 °C. The PCR product was separated by 1% agarose gel electrophoresis, and the gels were stained using ethidium bromide and viewed by UV transillumination.

### Western blot analysis

Cells were harvested and rinsed with PBS, and lyzed in denaturing lysis buffer (Applygen Technologies Inc. China) for 30 min on ice, centrifuged 12,000 g for 20 min at 4 °C. Protein concentrations were determined by BCA assay. Equal quantities (30 μg of protein) of cell extract were resolved by 10% SDS-PAGE, the resolved protein were electrophoretically transferred to PVDF membrane, and blocked with 5% fat-free dry milk in TBST for 1 h at room temperature. The membrane was immunoblotted with anti-γ**-**H2AX, anti-p21, anti-p-ATM, anti-ATM, anti-p-Mdm2, anti-Mdm2, anti-p-p53, anti-AKT, anti-β-actin (Cell Signaling Technology, USA), anti-p53, anti-Bax and anti-Bcl-2 (Santa Cruz, USA) antibodies in 5% milk TBST, at 4 °C overnight. The membranes were washed 3 times, incubated with HRP-conjugated secondary antibodies for 1 h at room temperature, and washed extensively before detection. The membranes were subsequently developed using ECL (FujiFilm, Japan) reagent (Applygen Technologies Inc. China) and exposed to film according to the manufacturer’s protocol.

### Xenograft mouse model

MCF-7 xenografts were initially established in female BALB/c nude mice (Center of Experimental Animals, Chinese Academy of Medical Sciences) at 6–7 weeks of age and body weight of 18–20 g. The mice were implanted with 5 × 10^6^ MCF-7 cells by subcutaneous injection into the interscapular area. Xenografts were maintained for two generations by subcutaneous implantation of about 50 mg non-necrotic tumor tissue using a trocar [20]. Tumor volume (mm^3^) was calculated by the formula, V = 1/2ab^2^, in which “a” and “b” represents length and width of tumor in mm. Relative tumor volume was calculated by using the formula: Relative tumor volume = Tx (absolute tumor volume of the respective tumor on day x) × 100/T0 (absolute tumor volume of same tumor on day0, when the treatment started). Drug treatment was started when the tumor size reached to above 100 mm^3^. The nude mice with xenografts were divided into 4 groups randomly. Each group contained 7 mice and was treated with various regimens on day 1. A dose of 23 mg/kg VP16 was administered on day 1 after grouping the mice and then every other day for one time, this group used as positive control. A group of nude mice was only treated with sterile normal saline as normal control. Two dosages of 2, and 4 mg/kg XWL-1-48 dissolved in sterile normal saline were orally given from day 1, then every other day for 4 weeks. All agents were orally administrated (o.p) in a volume of 0.2 ml/20 g body weight. The curve of tumor growth was drawn according to relative tumor volume and treatment time. In addition, tumors were excised from the mice and weighted it. The rate of inhibition (IR) was calculated. At the end point, tumor tissue was snap frozen in liquid nitrogen and stored at − 80 °C.

### Molecular docking studies

As the crystal structure of the human TopoIIα remains unavailable, we generated a structure model for this enzyme on the Swiss-model server automated system [21], using the crystal structure of the TopoIIβ-DNA-VP16 complex (PDB code: 3QX3 [22]) as the template. With respect to TopoIIβ, Top2α showed a sequence identity of 47.62%, so it was modeled on the known crystal structure of TopoIIβ as obtained from the PDB database. The top-scoring models were used. DNA molecule was copied from the template and included in the homology models. Docking studies with GOLD software version 5.2 [23] were carried out to get insights on the detailed interactions of TM with TopoIIα. The active site was defined by a sphere of 5.0 Å from the ligand EVP1 from the crystal structure of TopoIIβ (PDB code: 3QX3). The ligand used for the docking studies was constructed and prepared in SYBYL X2.1, and the energy was minimized using the external Tripos force field. The docked poses were scored using CHEMPLP scoring function. The best-docked pose of the ligand was visualized using Pymol Version 1.3.

### Immunofluorescence microscopy

MCF-7 cells were plated onto poly-L-lysine coated cover slips in 6-well plates. For analysis, the cells subjected to different treatments were fixed with 4% paraformaldehyde at room temperature for 15 min, washed three times with PBS and permeabilized in PBS containing 0.1% Triton X-100 for 10 min. The cover slips were washed three times with PBS again and then blocked in 3% normal goat serum for 2 h with shaking. The cells were counter-stained with PI or DAPI. Cover slips were then washed three times with PBS mounted onto slides using fluorescent mounting medium. Intensity changes in the γ-H2AX were imaged with an Olympus FV1000 (Olympus, Tokyo, Japan).

### Statistical analysis

Data were shown as mean ± SD. Statistical analysis of the data was performed using the one-way ANOVA by SPSS software. *p* < 0.05 was considered statistically significant.

## Results

### Effect of XWL-1-48 on TopoII activity and growth of breast cancer cells

TopoII is well validated as a target of anticancer drugs, and some agents targeting to TopoII are currently used clinically for treatment of various types of cancer. XWL-1-48 is a novel derivative of podophyllotoxin. Based on the chemical structure of XWL-1-48, we firstly evaluated the ability of XWL-1-48 binding to TopoII by using molecular docking software. As shown in Fig. 1b, c, the docking of XWL-1-48 on TopoII showed that two strong hydrogen bonds with distances of 2.7 Å and 2.9 Å were formed between XWL-1-48 and active residues (D436 and Q732, respectively). Furthermore, there were some potential pi-pi interactions between the phenyl ring of XWL-1-48 and DNA bases (dA12 and dG13). All these interactions support the potential binding of XWL-1-48 and TopoII. Accordingly, the effect of XWL-1-48 on TopoII activity was determined by kDNA-decatenation assay. kDNA-decatenation results indicated that XWL-1-48 significantly inhibited the TopoII activity in a concentration-dependent manner (Fig. 1d). Noticeably, the inhibitory activity of XWL-1-48 on TopoII is stronger than that of GL331. To investigate the cytotoxic activity of XWL-1-48, MCF-7 and MDA-MB-231 cells were treated with different concentrations of XWL-1-48 for 72 h. Cell proliferation was determined by the MTT assay. As shown in Fig. 1e, XWL-1-48 significantly inhibited the proliferation of MCF-7 and MDA-MB-231 cells in a concentration-dependent manner. Noticeably, more potent cytotoxicity of XWL-1-48 than that of its congener GL331 was observed, and the IC_50_ value is 0.40 ± 0.21 and 0.97 ± 0.49 μM, respectively. In addition to the MTT assay, we utilized the clonogenic assay to determine the effect of XWL-1-48 on MCF-7 and MDA-MB-231 clonogenic growth (Fig. 1f). XWL-1-48 significantly decreased colony number in a dose-dependent manner at low nM level.

### Effect of XWL-1-48 on ROS generation and DNA damage

**Fig. 2:**
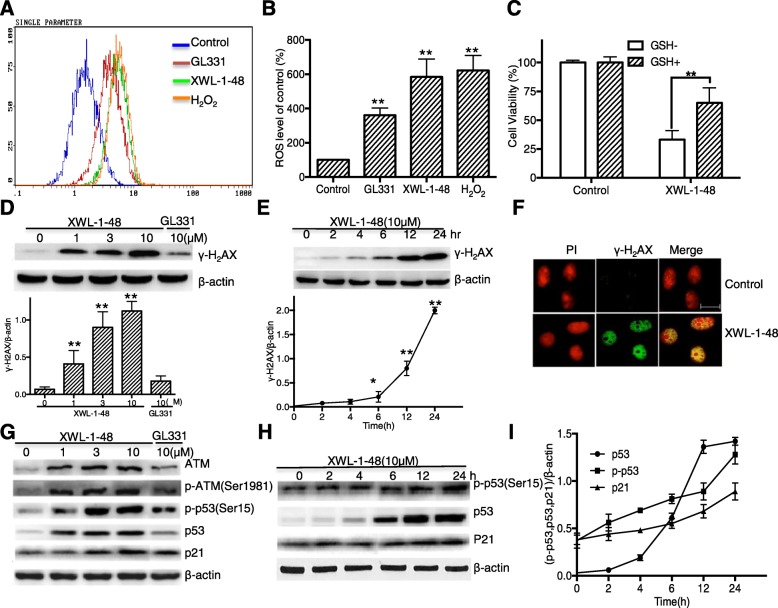
Effect of XWL-1-48 on ROS production and DNA damage, **a**, **b** After the cells treated with desired concentrations of XWL-1-48, GL331 and H_2_O_2_ for 24 h, ROS production was determined by flow cytometry. Columns represent the mean ± SD values obtained from three individual experiments; **c** MCF-7 cells were pre-treated with or without GSH (5 mM) for 1 h and then with XWL-1-48 (10 μM) for 24 h. The cell viability was determined by the MTT assay; **d** MCF-7 cells were treated with various concentration of XWL-1-48 or GL331 for 24 h, induction of γ-H2AX was determined by western blot; **e** MCF-7 cells were incubated with XWL-1-48 (10 μM) for indicated time point, γ-H2AX expression was measured; **f** Immunofluorescent staining of γ-H2AX in MCF-7 cells. Cells were incubated in 6-well plate overnight and then exposed to XWL-1-48 (1 μM) for 24 h. Cells were incubated with γ-H2AX antibody followed by incubation of secondary anti-rabbit IgG-FITC antibody (green). The nucleus was stained with PI (red). Images were captured using fluorescence microscope. Scale bar, 25 μm. **g** XWL-1-48 induced DNA damage by triggering ATM-related signaling pathways in MCF-7 cells. Expression levels of ATM, p-ATM, p-p53(ser15), p53 and p21 were analyzed. **h**, **i** MCF-7 cells were incubated with XWL-1-48 (10 μM) for the indicated time-point, the expression level of p-p53(ser15), p53 and p21 were determined by immunoblot. Data were shown as mean ± SD of three independent experiments. *p < 0.05, **p < 0.01 vs. control, respectively

As a new DNA poison, XWL-1-48 significantly inhibited cell growth and TopoII activity. By the way, generation of ROS and oxidative DNA damage is one of key mechanisms of DNA poisons [24]. With this in mind, the level of intracellular ROS was measured by flow cytometry. When the cells were incubated with GL331 (10 μM), XWL-1-48 (10 μM) and H_2_O_2_ (1 mM) for 24 h, the level of intracellular ROS was increased 3.6, 5.8, 6.2-fold compared to the control, respectively (Fig. 2a, b). Next, the cell viability was measured when MCF-7 cells were incubated with GSH (5 mM), a ROS scavenger, for 1 h and then treated with XWL-1-48 (10 μM). Figure 2c showed that pretreatment with GSH rescued cells from the cytotoxic effects of XWL-1-48, suggesting that the cytotoxic effect of XWL-1-48 in human breast cancer cells was ROS-dependent. The generation of ROS induces oxidative DNA damage, and led to cell death, we further investigated the effect of XWL-1-48 on DNA damage response. MCF-7 cells were incubated with XWL-1-48 for 24 h, γ-H2AX, a known DNA damage marker, was determined by immunoblot. As shown in Fig. 2d, XWL-1-48 induced γ-H2AX expression in a concentration-dependent manner. 1 μM of XWL-1-48 significantly elevated γ-H2AX. Moreover, XWL-1-48 led to an increase of γ-H2AX in a time-dependent. After exposure to XWL-1-48 for only 6 h, the expression of γ-H2AX was significantly increased (Fig. 2e). Meanwhile, induction of γ-H2AX was observed by fluorescent staining (Fig. 2f). In addition to γ-H2AX, we further determined the effect of XWL-1-48 on DNA damage pathway ATM/p53/p21. After incubated with XWL-1-48 (1, 3, 10 μM) for 24 h, the expression of p-ATM, ATM, p-p53, p53 and p21 were detected by immunoblot analysis. The expression level of p-ATM, p-p53, p53 and p21 was increased. 1 μM of XWL-1-48 effectively induced p-ATM and p53 expression (Fig. 2g). Meanwhile, we also investigated the induction of p-p53, p53 and p21 by XWL-1-48 at different time point. The expression level of p-53, p53 and p21 were significantly increased after 12 h treatment (Fig. 2h, i). Thus, ROS generation and DNA damage is responsible to the cytotoxicity of XWL-1-48.

### Effect of XWL-1-48 on cell cycle

**Fig. 3:**
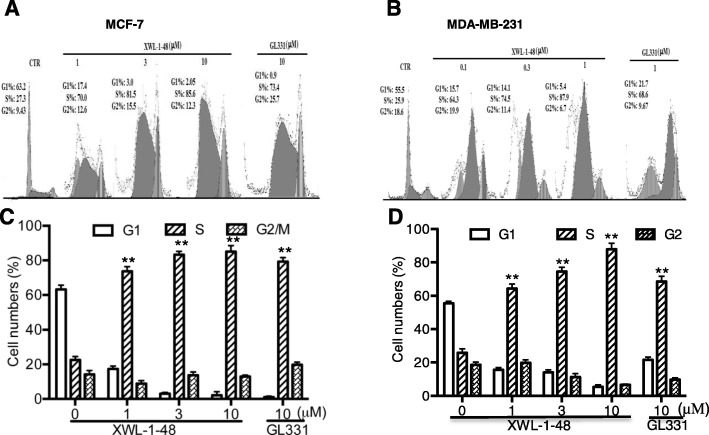
Effect of XWL-1-48 on cell cycle in breast cancer. MCF-7 (**a**, **c**) or MDA-MB-231 (**b**, **d**) cells were treated with XWL-1-48 (1, 3, 10 μM) and GL331 (10 μM) for 24 h, then cells were harvested, fixed and stained with PI for flow cytometry; Data were shown as mean ± SD of three independent experiments

DNA damage can lead to cell cycle arrest. We next performed flow cytometry to examine the effect of XWL-1-48 on cell cycle, MCF-7 and MDA-MB-231 cells were treated by XWL-1-48 (1, 3, 10 μM) and GL331 (10 μM) for 24 h, and cell cycle distribution was measured. As seen in Fig. 3, S phase was significantly blocked in MCF-7 and MDA-MB-231 cells.

### Effect of XWL-1-48 on apoptosis of breast cancer

**Fig. 4:**
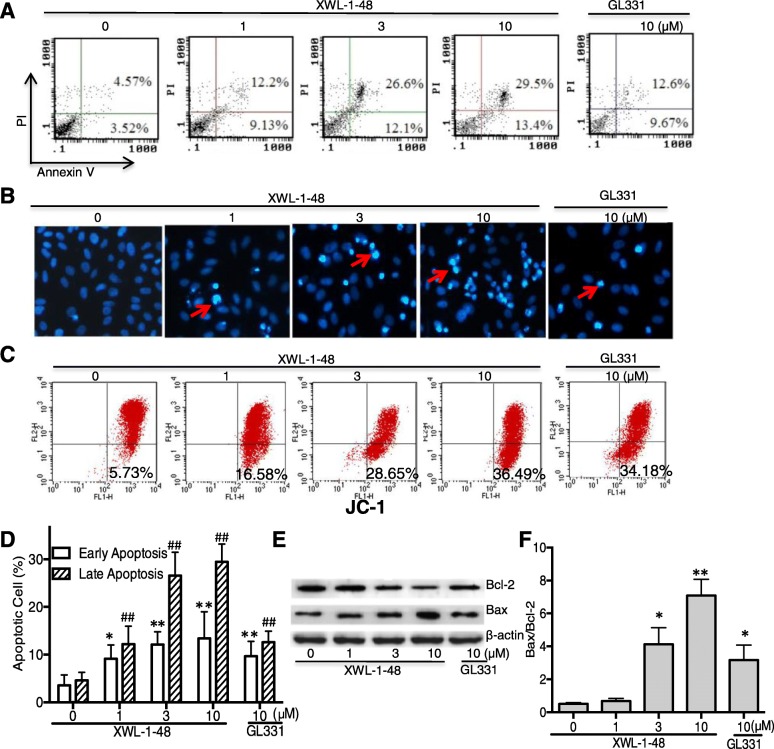
Effect of XWL-1-48 on cellular apoptosis of breast cancer. **a**, **d** Treatment with XWL-1-48 (1, 3, 10 μM) and GL331 (10 μM) for 24 h, early and late apoptotic cells were detected by flow cytometry. A representative of three independent experiments is shown; **b** Apoptosis of MCF-7 cells was detected by DAPI staining. MCF-7 cells were treated by XWL-1-48 or GL331 for 24 h, then the cells harvested, fixed and stained with 4, 6-diamidino-2-phenylindole (DAPI); **c** Loss mitochondrial membrane potential of cells treated by XWL-1-48 or GL331. MCF-7 cells treated with or without XWL-1-48 and GL331 for 24 h at indicated concentrations. The cells were harvested and washed, and then cells were incubated for 20 min in freshly prepared JC-1 solution at 37 °C. Spare dye was removed by dye buffer solution washing and cell-associated fluorescence was measured by flow cytometry; **e**, **f** MCF-7 cells were incubated with XWL-1-48 (1, 3, 10 μM) and GL331 (10 μM) for 24 h, expression of Bcl2, Bax were determined by western blot analysis. A representative result is shown from at least three independent experiments. Data were shown as mean ± SD of three independent experiments, *, *p* < 0.05, **, *p* < 0.01 or #, *p* < 0.05, ##, *p* < 0.01 vs. control group, respectively

Due to the potent cytotoxic activity and evidently ROS-mediated DNA damage, it suggests that XWL-1-48 could induce cellular apoptosis in breast cancer. Apoptosis may be responsive to XWL-1-48-mediated anticancer activities. Accordingly, the ability of XWL-1-48 on inducing apoptosis in MCF-7 cells was analyzed by using AnnexinV/PI and DAPI staining. As shown in Fig. 4a, d, XWL-1-48 resulted in an increase of early and late apoptotic cells in a dose-dependent manner. The ability of XWL-1-48 inducing apoptosis is stronger than that of GL331. Meanwhile, we also focused on the morphological changes of apoptosis using DAPI staining. As seen in Fig. 4b, MCF-7 cells with normal morphology were observed in control group, whereas MCF-7 cells with fragmented chromatin and apoptotic bodies were noted following treatment with XWL-1-48. These results suggest that XWL-1-48 is capable of inducing marked apoptotic morphological changes in MCF-7 cells. Given that the collapse of the mitochondrial membrane potential is an early step in the induction of apoptosis by the mitochondrial pathway. Therefore, the variation of mitochondrial membrane potential was determined by JC-1 staining analysis in MCF-7 cells. In non-apoptotic cells the dye accumulates and aggregates within the mitochondria, resulting in bright red staining. In apoptotic cells, due to the collapse of the membrane potential, the JC-1 cannot accumulate within the mitochondria and remains in the cytoplasm in its green-fluorescent monomeric form. As shown in Fig. 4c, treatment with XWL-1-48 (1, 3, 10 μM) for 24 h led to 16.6%, 28.7% and 38.5% cell membrane potential collapse, respectively. Bcl-2 family proteins play a major role in the control of mitochondria-mediated intrinsic apoptosis [25, 26]. The expression of anti-apoptotic protein Bcl-2 and pro-apoptotic protein Bax were determined by immunoblot. Treatment with XWL-1-48 significantly reduced the expression of Bcl-2 and enhanced the expression of Bax. The relative ratio of Bax and Bcl-2 significantly elevated (Fig. 4e, f). These results suggest that XWL-1-48 effectively induced mitochondrial apoptosis in breast cancer cells.

### Effect of XWL-1-48 on PI3K/Akt/Mdm2 pathway

**Fig. 5:**
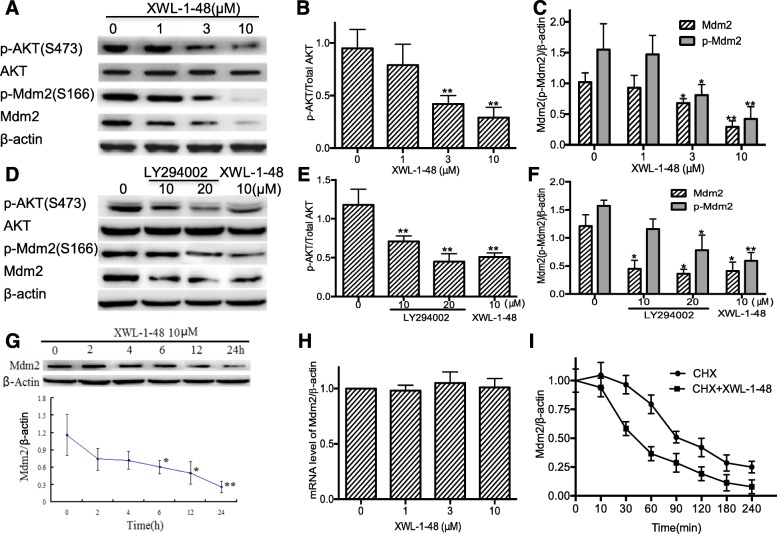
Effect of XWL-1-48 on PI3K/Akt/Mdm2 pathway. **a**-**c** MCF-7 cells were treated by XWL-1-48 (1, 3, 10 μM) for 24 h, protein expression of p-Akt, Akt, p-Mdm2 and Mdm2 were measured by Western blot analysis. The changes of p-Akt/Akt and p-ATM/ATM were quantified using Image J software; **d**-**f** MCF-7 cells were exposure to a known pan-PI3K inhibitor LY294002 or XWL-1-48 for 24 h, protein expression level of p-Akt, Akt, p-Mdm2 and Mdm2 was determined using immunoblot; **g** After treated with XWL-1-48 (10 μM) for 0, 2, 4, 6, 12, 24 h, cells were harvested. The protein expression level of Mdm2 was analyzed by western blot; **h** After incubated with XWL-1-48 (1, 3, 10 μM) for 24 h, the mRNA expression of Mdm2 was detected by RT-PCR; **i** MCF-7 cells were exposed to 100 μg/ml cycloheximide (CHX, known as protein synthesis inhibitor) with or without XWL-1-48 (10 μM) for 0, 10, 30, 60, 90, 120, 180 and 240 min to block protein synthesis. The cells were collected for Western blot analysis. A representative result is shown from at least three independent experiments. *p < 0.05, **p < 0.01 vs. control, respectively

p53 is activated in response to DNA damage, lead to induction of apoptosis and inhibition of cancer cell growth [27]. Mdm2 play a key role in p53-mediated signaling pathway. Interestingly, there is crosstalk between Akt and the p53 pathway. DNA damage leads to an irreversible apoptotic event, activation of p53 may contribute to apoptosis by inhibition of Akt [28]. Meanwhile, in the presence of appropriate survival signals, Akt activation may lead to Mdm2 phosphorylation. Based on these findings, we investigated the effect of XWL-1-48 on PI3K/Akt/Mdm2 pathway. As seen in Fig. 5a-c, XWL-1-48 significantly suppressed activation of Akt and decreased expression of p-Mdm2 and total Mdm2. Similar inhibitory activity of LY294002, a known pan-PI3K inhibitor, on PI3K/Akt/Mdm2 pathway was observed (Fig. 5d-f). Blockage of PI3K/Akt downregulated the expression of p-Mdm2 and total Mdm2. MCF-7 cells were incubated with XWL-1-48 for 0, 2, 6, 12 and 24 h, expression of Mdm2 was also determined by immunoblot. XWL-1-48 resulted in a decrease of Mdm2 in a time-dependent manner (Fig. 5g). After exposure to XWL-1-48 for only 6 h, Mdm2 expression was significantly reduced to 50%. To understand the regulation mechanism of Mdm2 by XWL-1-48, we further studied the effect of XWL-1-48 on transcription level of Mdm2 using RT-PCR. Our result showed that XWL-1-48 did not affect mRNA expression of Mdm2 (Fig. 5h). Above results indicated that XWL-1-48 significantly inhibited Mdm2 expression but did not affect the transcriptional level of Mdm2. It suggests that XWL-1-48 might enhance Mdm2 degradation. To test this possibility, we determined the half-life of Mdm2 protein by the introduction of cycloheximide (CHX), a known protein synthesis inhibitor. In presence of XWL-1-48, the Mdm2 protein had a shorter half-life (45 min) than that (90 min) of the control (Fig. 5i).

### In vivo biological activity of XWL-1-48 on the growth of breast cancer

**Fig. 6:**
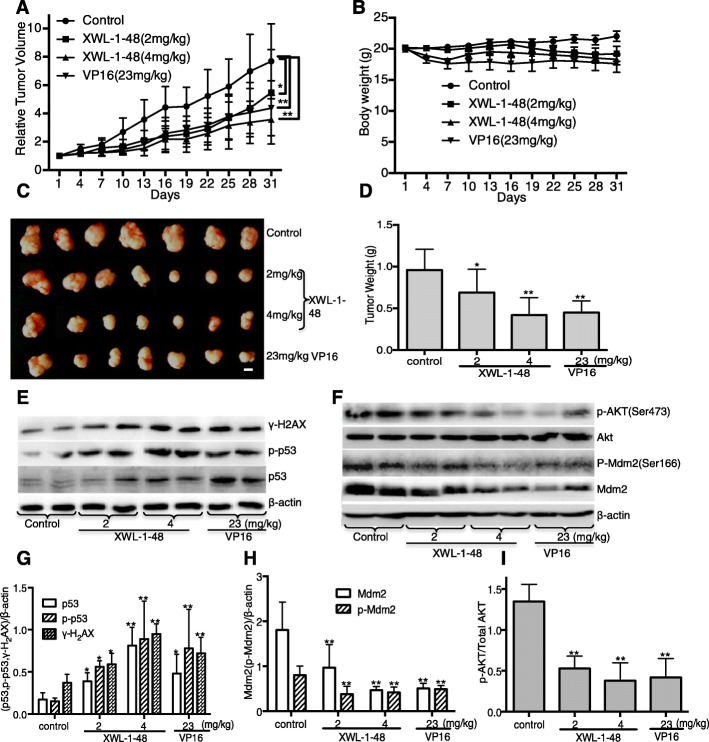
Effect of XWL-1-48 on growth of breast cancer in vivo. Athymic nude mice bearing MCF-7 tumor xenografts were orally administered every other day vehicle control (sterile normal saline), XWL-1-48 (2 and 4 mg/kg), and VP16 (23 mg/kg) for 30 days. **a** Relative Tumor Volume and (**b**) body weight were measured every other day; **c** The picture showed the tumor size of MCF-7 xenograft at the end of the experiment; **d** Tumor tissues were weighed and further calculated inhibitory rate of tumor weight. Data are mean ± SD of the tumor weight for each group of 7 experimental animals; **e**-**i** Protein expression in xenografts was determined after 30-day of XWL-1-48 or VP16 treatment. Tumors were harvested 2 h after the last dose. γ-H2AX, p-p53, p53; p-Akt, Akt, p-Mdm2 and total Mdm2 were analyzed by western blot, further quantified by image J. A representative result is shown from at least three independent experiments. *p < 0.05, **p < 0.01 vs. control, respectively

Given that the potent cytotoxic activity and strong ability of inducing ROS-mediated DNA damage in breast cancer cells, we further evaluated in vivo antitumor activity of XWL-1-48 using mice bearing MCF-7 xenograft. Mice were orally administered different doses of XWL-1-48 (2, 4 mg/kg) every other day for 4 weeks. At the end of the treatment period, tumor volume was decreased 34.2 and 58.7%, respectively, as compared with vehicle-treated mice (Fig. 6a). Inhibition of high dose XWL-1-48 on tumor growth is more potent than that of VP16 (23 mg/kg). After treated by XWL-1-48 (4 mg/kg) and VP16 (23 mg/kg) for 1 week, the body weight of treated mice was decreased 12.0% (Fig. 6b). It suggests that XWL-1-48 (4 mg/kg) and VP16 (23 mg/kg) cause some toxicity response. Meanwhile, the tumor weight was also measured at the end of experiment. Inhibitory rate of tumor weight in 2 and 4 mg/kg XWL-1-48-treated group was 28.0 and 56.0%, respectively (Fig. 6c, d). Tumor tissue was obtained on day31, 2 h following XWL-1-48 orally administration, and protein expression was determined by western blot analysis. Administration of XWL-1-48 to the mice resulted in a dose-dependent activation of H2AX and p53 in the xenograft tumors (Fig. 6e, g). In addition, expression levels of key protein in PI3K/Akt/Mdm2 pathways were investigated. We observed potent suppression of p-AKT, p-Mdm2 and Mdm2, which correlated with our in vitro results (Fig. 6f, h, i).

## Discussion

Topoisomerases II (TopoII) are a well-validated target for treatment of cancer. In eukaryotic cells, there are two isoforms of TopoII, α and β, create DNA double-stranded breaks (DSBs) to allow the passage of a second double-stranded DNA, correct topological DNA errors in replication, transcription, recombination, and chromosome condensation and decondensation [29]. Although the biological function of TopoII is a key role for ensuring genomic integrity, the ability to targeted inhibition of TopoII and trigger DNA damage is a successful strategy for cancer chemotherapy [10]. TopoII poisons (e.g. VP16, teniposide, doxorubicin, and mitoxantrone) are frontline therapies for a variety of cancers, including breast cancer [30]. In the present study, XWL-1-48 was identified as a novel orally TopoII poison against breast cancer. Noticeably, XWL-1-48 exhibited better water solubility and more potent activity than VP16 and GL331 for oral administration.

Under the condition of genotoxic stress (for example, TopoII poisons), cells activate a whole signaling network, termed as DNA damage response (DDR) which senses the damage and coordinates multiple pathways that either arrest the cell cycle or induce apoptosis [31]. Inactivation of DDR plays an important role in tumor progression. ATM (AT mutated) and ATR (ATM and Rad3-related) have a central role in coordinating the DDR, including control of cell cycle, DNA repair and apoptosis [32]. Among targets of ATM and ATR is p53, which has a key role in controlling DNA damage-induced G1/S and G2/M checkpoints [33]. Herein, we found that XWL-1-48 significantly activated ATM/p53/p21 pathway, arrested cell cycle at S phase. During the process of DNA damage, double strand break (DSB) can rapidly induce the phosphorylation of H2AX, hundreds to thousands of γ-H2AX molecules gather and surround the DSB site to form foci both to keep the chromatin open and to serve as a platform for the following DNA damage response. Therefore, the phosphorylated histone H2AX (γ-H2AX) can be used biomarker for DNA double-strand breaks. In the present study, XWL-1-48 significantly induced γ-H2AX in a time- and dose-dependent manner. Our result suggests that treatment of XWL-1-48 led to a classic DNA damage.

In addition, in the presence of DNA poisons, the generation of ROS promotes the permeability transition pore complex (PTPC) opening. Extreme quantities of ROS can induce damage to lipids, and proteins, leading to DNA oxidative damage and resulting in cell death [34–37]. Oxidative DNA damage can prompt the activity of Bax proteins, a family of pro-apoptotic Bcl2 members, causing penetrability of the mitochondrial membrane [34]. This event subsequently gives rise to the activation of caspase cascade, which ultimately resulting in apoptotic features, such as DNA condensation and fragmentation, and membrane blabbing. Our data provide evidence that XWL-1-48 led to increase of ROS generation, loss of DCm in MCF-7 cells. We observed that apoptosis was induced by XWL-1-48 in a dose-dependent manner. Treatments with XWL-1-48 significantly led to an increase of Bax and decrease of Bcl-2, elevated the ratio of Bax/Bcl-2, loss the potential of mitochondrial membrane, and finally activated caspase-3.

Recently, it has reported that PI3K/Akt pathway is associated with p53-mediated transcription and apoptosis. Activation of Akt enhances the ubiquitination-promoting function of Mdm2 by phosphorylation of Ser^186^, which results in reduction of p53 protein [38]. Given that the effect of XWL-1-48 on activation of p53, we further investigated the effect of PI3K/Akt/Mdm2 pathway. Herein, we confirmed by Western blot that treatment with XWL-1-48 resulted in reduced phosphorylation of Akt and Mdm2, and increased phosphorylation of p53, which were all important proteins in the p53 pathway. Inhibition of Mdm2 in a dose- and time-dependent manner was observed in XWL-1-48 treatment breast cancer. Further studies showed that XWL-1-48 prompted degradation of Mdm2 through reduced the half-life of Mdm2.

## Conclusions

**Fig. 7:**
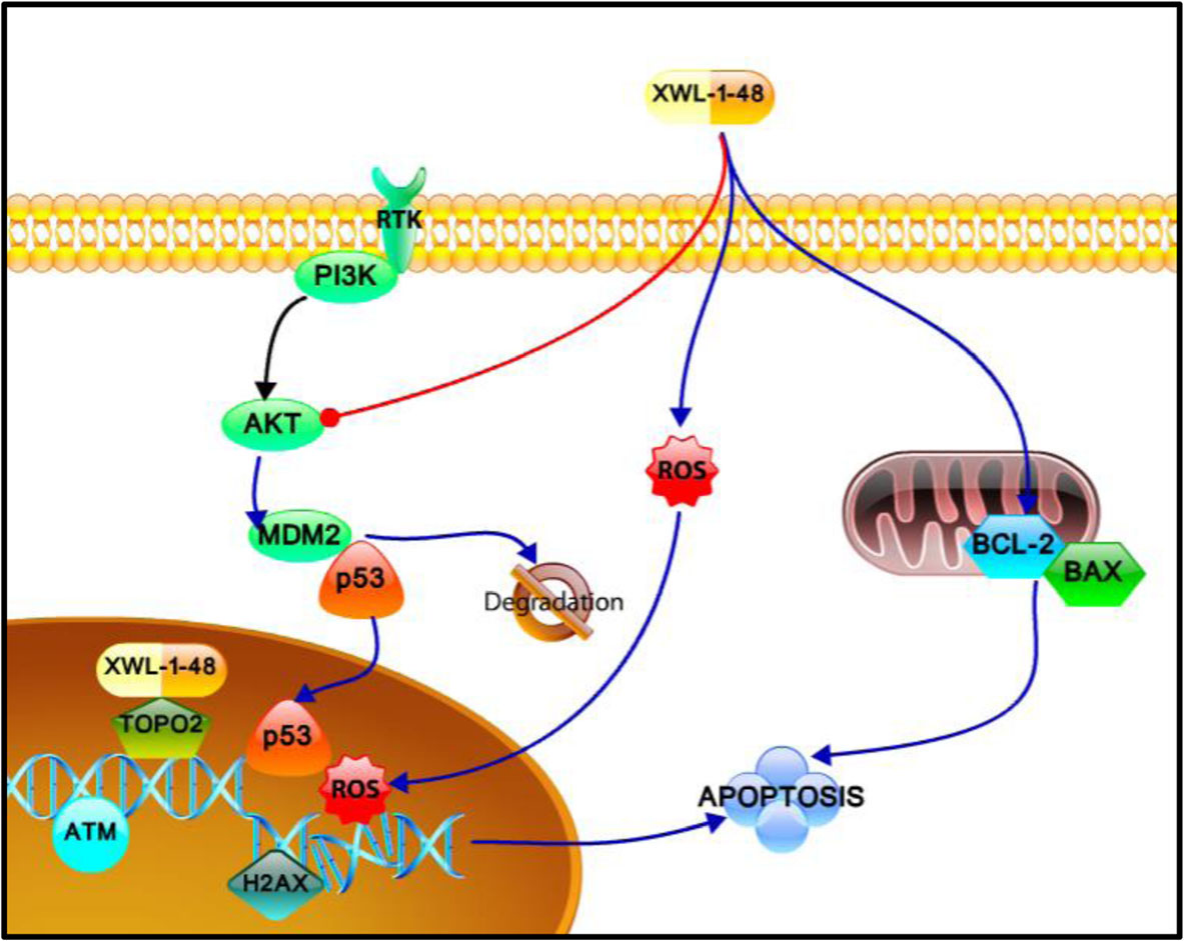
Mechanism of XWL-1-48 inhibits growth of breast cancer cells. XWL-1-48, a novel orally topoisomeraseII inhibitor, exerts potent in vitro and in vivo anti-tumor activity against breast cancer. Treatment with XWL-1-48 significantly inhibited TopoII activity, triggered DNA damage response, activated ATM/p53/p21 pathways, arrested cell cycle at S phase, and induced mitochondria-mediated apoptosis. Meanwhile, XWL-1-48 strongly blocked PI3K/Akt/Mdm2 pathway, prompted degradation of Mdm2, and suppressed breast cancer cell survival

In summary, XWL-1-48 significantly suppressed TopoII activity through directly binding to the enzyme, triggered ROS production and DNA damage, arrested S-phase, and induced intrinsic apoptosis. Meanwhile, XWL-1-48 evidently blocked PI3K/Akt/Mdm2 pathway, enhanced degradation of Mdm2, and suppressed breast cancer cell survival (Fig. 7). Our results suggest that XWL-1-48 serving as a potential TopoII inhibitor for the treatment of breast cancer.

## Abbreviations

(DSB): Double strand break
AKT: Protein kinase B
DCm: Mitochondrial transmembrane potential
DDR: DNA damage response
MDM2: Mouse double minute 2 homolog
PI3K: Phosphatidylinositol-4, 5-bisphosphate 3-kinase
PTPC: Permeability transition pore complex
ROS: Reactive oxygen species
SD: Standard deviation
TopoII: Topoisomerase II
